# No aftereffects of high current density 10 Hz and 20 Hz tACS on sensorimotor alpha and beta oscillations

**DOI:** 10.1038/s41598-021-00850-1

**Published:** 2021-11-01

**Authors:** Louis-Philippe Lafleur, Audrey Murray, Manon Desforges, Kevin Pacheco-Barrios, Felipe Fregni, Sara Tremblay, Dave Saint-Amour, Jean-François Lepage, Hugo Théoret

**Affiliations:** 1 Département de Psychologie, Université de Montréal, CP 6128. Succ. Centre-Ville, Montréal, QC H3C 3J7 Canada; 2 Département de Psychologie, Université du Québec à Montréal, Montréal, Canada; 3 Centre de Recherche du Centre Hospitalier Universitaire Sainte-Justine, Montréal, Canada; 4 Centre Intégré Universitaire de Santé et de Services Sociaux du Nord-de-l’île-de-Montréal, Montréal, Canada; 5 Département de Psychoéducation et de Psychologie, Université du Québec en Outaouais, Gatineau, Canada; 6 University of Ottawa Institute of Mental Health Research at The Royal, Ottawa, Canada; 7 Neuromodulation Center and Center for Clinical Research Learning, Spaulding Rehabilitation Hospital and Massachusetts General Hospital, Harvard Medical School, Boston, MA USA; 8 Département de Pédiatrie, Faculté de Médecine et des Sciences de la Santé de l’Université de Sherbrooke, Centre de Recherche du CHU Sherbrooke, Sherbrooke, Canada

**Keywords:** Excitability, Motor cortex

## Abstract

Application of transcranial alternating current stimulation (tACS) is thought to modulate ongoing brain oscillations in a frequency-dependent manner. However, recent studies report various and sometimes inconsistent results regarding its capacity to induce changes in cortical activity beyond the stimulation period. Here, thirty healthy volunteers participated in a randomized, cross-over, sham-controlled, double-blind study using EEG to measure the offline effects of tACS on alpha and beta power. Sham and high current density tACS (1 mA; 10 Hz and 20 Hz; 0.32 mA/cm^2^) were applied for 20 min over bilateral sensorimotor areas and EEG was recorded at rest before and after stimulation for 20 min. Bilateral tACS was not associated with significant changes in local alpha and beta power frequencies at stimulation sites (C3 and C4 electrodes). Overall, the present results fail to provide evidence that bilateral tACS with high current density applied over sensorimotor regions at 10 and 20 Hz reliably modulates offline brain oscillation power at the stimulation site. These results may have implications for the design and implementation of future protocols aiming to induce sustained changes in brain activity, including in clinical populations.

## Introduction

The endogenous oscillations of the brain are associated with specific cognitive functions and are believed to play an important role in regimenting communication between cortical and subcortical areas^1,2^. Considering their putative association with various cognitive states and pathophysiological disorders^3,4^, there is high interest in modulating these oscillations non-invasively. Among non-invasive brain stimulation tools, transcranial alternative current stimulation (tACS) appears particularly well-suited for this task, as it consists in the application of a weak (< 2 mA) sinusoidal electric current passing through the scalp that entrains cortical oscillations at a specific frequency in the nearby cortex^5,6^.

Several studies have shown that tACS can modulate cognition^7^, perception^8^, corticospinal excitability^9^ and cortical oscillations^6^ during the stimulation period (*on-line* effects). For example, studies using transcranial magnetic stimulation (TMS) have shown frequency-specific tACS effects on corticospinal excitability^9–11^. Similarly, administration of tACS in the alpha band (8–12 Hz) to the occipital and parietal cortex has been shown to entrain oscillations in this frequency range^6,12^. Furthermore, the immediate effects of tACS are not restricted to the vicinity of the stimulated sites, as changes in resting-state fMRI connectivity have been reported during alpha^13,14^, beta^15^ and gamma^16^ tACS.

While the neurophysiological consequences of online tACS may explain its documented influence on behavior^17–22^, one outstanding question is whether tACS can induce changes that outlast the stimulation period (*off-line* effects). Although the precise mechanism of its action is not completely understood, it is believed that tACS-induced oscillation entrainment and neuronal synchronization may lead to lasting neuroplastic changes^5,12,23,24^. The most consistent finding for offline tACS has been that stimulation applied at alpha frequencies over occipital areas can increase alpha power^12,24–27^ for up to 70 min after the end of stimulation^28^. Recent studies, however, reported no aftereffect in the alpha band following alpha tACS over occipital^29,30^, parietal^31^, somatosensory^32^ and frontal^32^ cortex. Beta tACS has also been shown to increase beta power following stimulation of visual^27^ and parietal cortex^33^, although Berger and collaborators^34^ found no effect of 20 Hz stimulation on beta power.

Alpha and beta oscillations are known to be predominant in sensorimotor cortex, peaking at approximately 10 Hz and 20 Hz, respectively^35,36^. Studies using TMS to assess tACS-induced changes in corticospinal excitability have provided conflicting results. Whereas some studies have reported offline effects of alpha or beta tACS on corticospinal excitability^11,37,38^, most studies have shown no aftereffects^19,39–43^ or mixed results^21,44,45^.

The aftereffects of alpha and beta tACS in sensorimotor cortex have also been investigated using EEG and MEG, and here again results have been inconsistent. Wach and collaborators^46^ and Sugata et al.^47^ found no evidence for offline modulation of MEG alpha and beta oscillations following 10 Hz and 20 Hz tACS with almost identical stimulation parameters. In both of these studies, tACS was administered for ten minutes in a C3-supraorbital montage with an intensity of 1mA^46,47^. Similarly, a recent EEG study reported no aftereffects in the alpha and beta frequencies following 10 min of 10 Hz and 20 Hz tACS applied for 10 min in a C3-supraorbital montage at 1mA^48^. The induction of significant tACS aftereffects in sensorimotor cortex, however, has been reported in a recent study that used a different stimulation approach. Wischnewski and collaborators^49^ applied 20 Hz tACS using high-definition stimulation^50^, where a small round electrode (3.14 cm^2^) positioned over C3 was surrounded by four return electrodes at T7, F3, Cz and P3. Stimulation at 20 Hz was applied for 15 min at an intensity of 2 mA. Resting-state oscillations in the beta frequency were significantly increased for up to 60 min after the end of stimulation, and the effects were restricted to the stimulated frequency and electrode^49^.

Taking into consideration the fact that tACS modulation of resting-state EEG in sensorimotor cortex has been reported for high^49^ but not low^46–48^ current densities and that TMS evidence shows that tACS-induced changes corticospinal excitability are also dependent on current intensity and density^11,51^, the present study was designed to probe the effects of high current density tACS on sensorimotor alpha and beta oscillations. To this end, 10 Hz, 20 Hz, and sham stimulation were applied with a current density of 0.32 mA/cm^2^ in 30 healthy participants in a randomized, cross-over, sham-controlled, double-blind protocol. Stimulating electrodes (3.14 cm^2^) were positioned over C3 and C4 in a bilateral protocol that has been previously shown to induce aftereffects in alpha and beta frequencies^34,52,53^. Resting-state EEG was acquired with the same electrodes used for stimulation for 5 min prior to tACS and for 20 min after the end of stimulation.

## Results

### Participant blinding

Participants were asked if they believed they had received active or sham stimulation after each session. For all testing sessions, 72% of participants correctly identified sham stimulation, while 83% and 93% of participants correctly identified as « active » the 10 Hz and 20 Hz stimulation conditions, respectively. Cochran’s Q test did not reveal significant differences between conditions (χ^2^ (2) = 4.91; *p* = 0.086). To remove the bias of previous responses given by each participant through the sessions, an additional analysis was performed on the first visit data only. Nine participants out of ten correctly identified 10 Hz stimulation as active, 10 participants of ten correctly identified 20 Hz stimulation as active, and 7 participants of ten correctly identified sham stimulation as inactive at their first visit. Fisher’s exact test revealed no significant difference between groups for the proportion of correct answers (*p* = 0.29).

### Alpha and beta power

**Figure 1:**
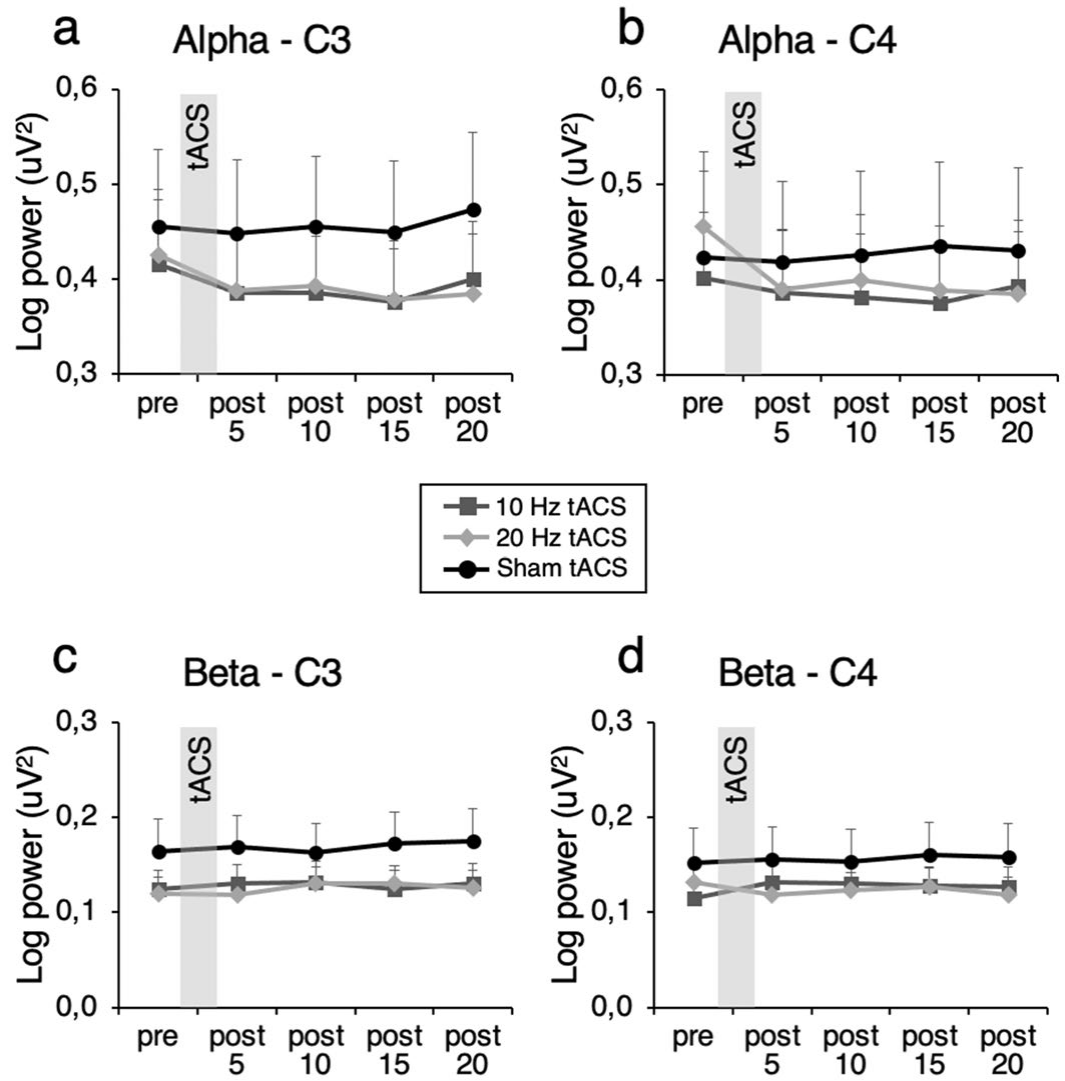
Effects of tACS on alpha and beta oscillations. Log-transformed alpha power at electrode (**a**) C3 and (**b**) C4 following stimulation. Log-transformed beta power at electrode (**c**) C3 and (**d**) C4 following stimulation. Error bars represent SEM.

For *α*-power at electrode site C3 (Fig. 1a), there was no main effect of *Condition* (*F*_1.69, 49.14_ = 1.70; *p* = 0.20; η^2^_partial_ = 0.06), no main effect of *Time* (*F*_2.15, 62.29_ = 2.35; *p* = 0.10; η^2^_partial_ = 0.08) and no *Condition* X *Time* interaction (*F*_4.39, 127.18_ = 0.83; *p* = 0.52; η^2^_partial_ = 0.03).

For *α*-power at electrode site C4 (Fig. 1b), there was no main effect of *Condition* (*F*_1.81, 52.52_ = 0.40; *p* = 0.66; η^2^_partial_ = 0.01), no main effect of *Time* (*F*_1.89, 54.87_ = 1.86; *p* = 0.17; η^2^_partial_ = 0.06) and no *Condition* X *Time* interaction (*F*_3.53, 102.25_ = 1.97; *p* = 0.11; η^2^_partial_ = 0.06).

For *β*-power at electrode site C3 (Fig. 1c), there was no main effect of *Condition* (*F*_1.34, 38.80_ = 1.87; *p* = 0.18; η^2^_partial_ = 0.06), no main effect of *Time* (*F*_2.20, 63.81_ = 0.84; *p* = 0.45; η^2^_partial_ = 0.03) of and no *Condition* X *Time* interaction (*F*_2.89, 83.77_ = 0.95; *p* = 0.42; η^2^_partial_ = 0.03).

For *β*-power at electrode site C4 (Fig. 1d), there was no main effect of *Condition* (*F*_1.43, 41.36_ = 1.03; *p* = 0.34; η^2^_partial_ = 0.03), no main effect of *Time* (*F*_1.66, 48.02_ = 0.29; *p* = 0.71; η^2^_partial_ = 0.01) and no *Condition* X *Time* interaction. (*F*_4.28, 124.24_ = 2.39; *p* = 0.05; η^2^_partial_ = 0.08).

**Figure 2:**
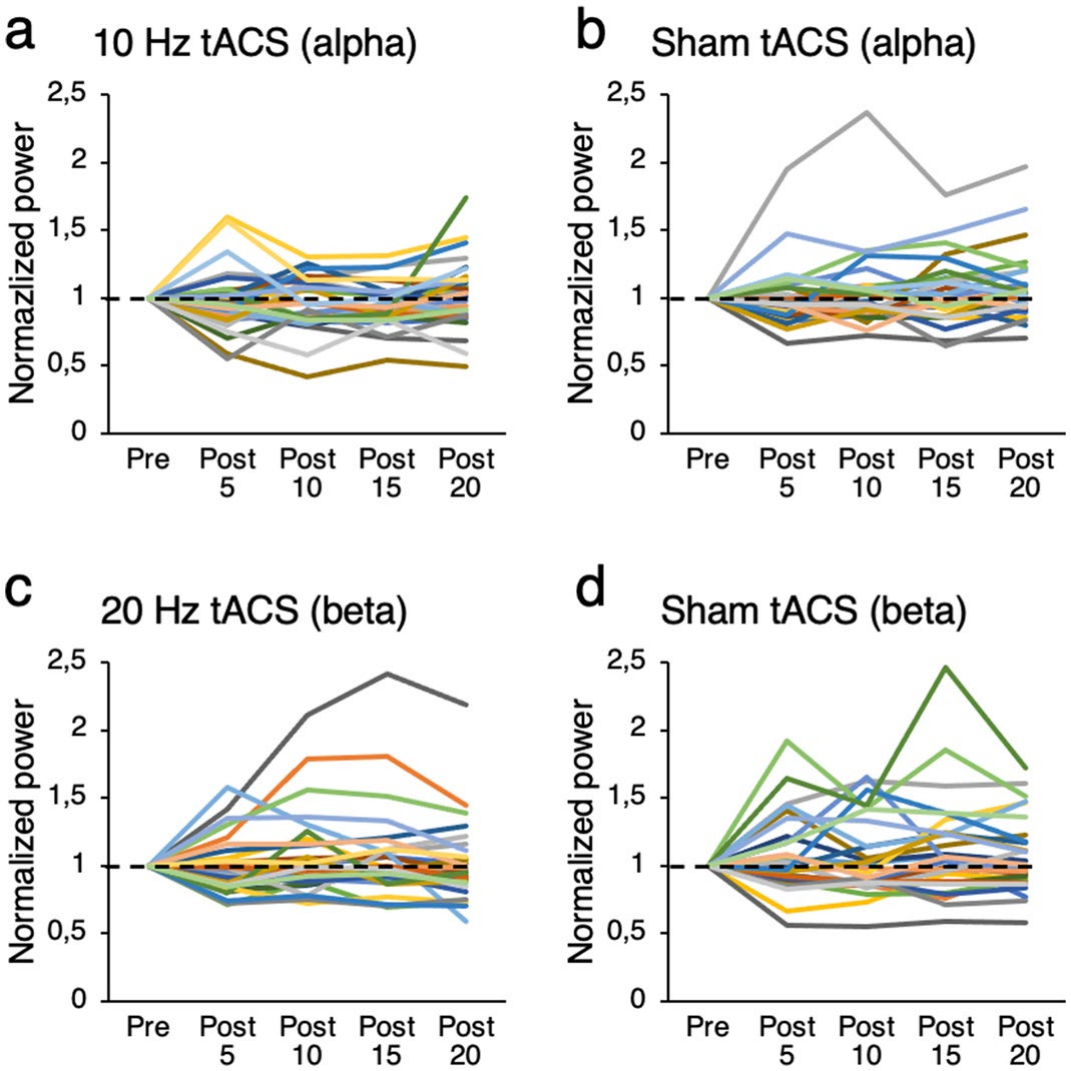
Individual response to tACS normalized to baseline values. Individual alpha and beta log-transformed power following 10 Hz, 20 Hz, or sham stimulation at electrode C3. Note that one participant with high power values is not presented in the figure for clarity but was included in the statistical analysis.

Pearson’s correlations were computed to determine if baseline alpha or beta activity was associated with the strength of the tACS effect in the alpha and beta frequencies. There was no statistically significant correlation between baseline alpha or beta power and tACS effects on post stimulation alpha ratios (T1/T0, T2/T0, T3/T0, T4/T0) at electrodes C3 and C4 (all *r* between -0.08 and -0.33 and all p > 0.07 for alpha; all *r* between -0.09 and -0.30 and all p > 0.10 for beta). Individual data normalized to pre-tACS values are shown in Fig. 2.

Bayesian repeated measures ANOVAs were also conducted to quantify the plausibility of both the null and the alternative hypotheses, permitting interpretation of null findings. In Bayesian inference, the likelihood of the data is considered under both hypotheses, and these probabilities are compared via the Bayes factor (BF). According to Lee and Wagenmakers’ classification^54^ (see also Stefan et al.^55^), the level of evidence is deemed inconclusive/anecdotal for BF between 0.33 and 3, moderate for BF < 0.33 or > 3, and strong for BF < 0.01 or > 10. Following the JASP guidelines, BF comparing the null model against all other models were computed and each experimental effect was obtained by calculating the inclusion BF across matched models. Results revealed relatively strong evidence supporting the null hypothesis (*H*_0_) over *H*_1_ for the *Condition X Time* interaction under both *α* and *β* frequencies and both electrodes C3 and C4 (all BF values were lower than 0.01 (from 0.003 to 0.006)). For more complete Bayesian analysis, see Table S1-S8, and Table S9 for a summary of the classification.

**Figure 3:**
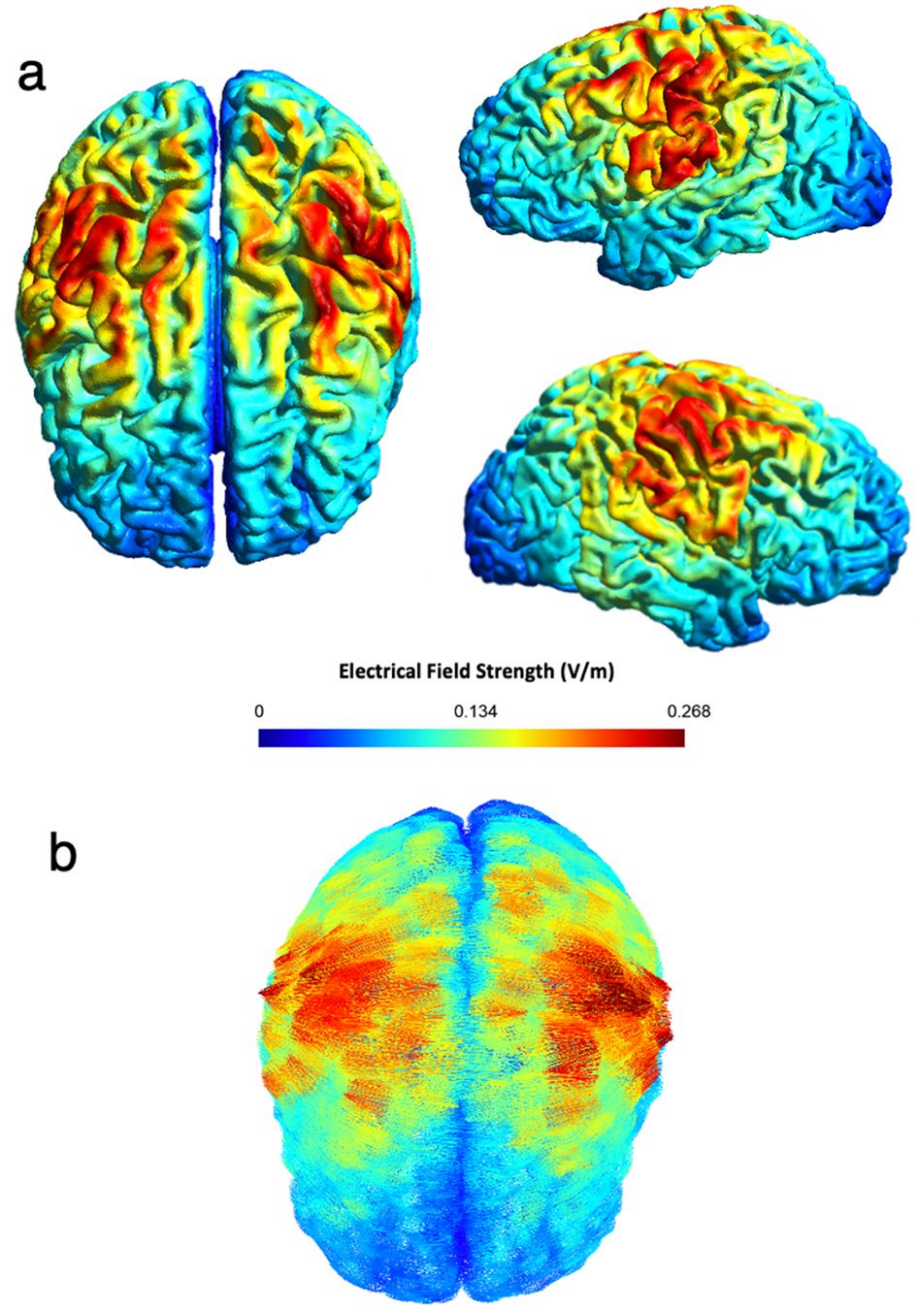
Electrical field model. (**a**) Normal component of the electrical field (electrical field strength in V/m) and its neuroanatomical distribution. (**b**) Representation of the electrical field vectors for the same model results.

Electrical field simulation of the tACS protocol using the Finite-Element Method (FEM) showed focal and strong fields occurring bilaterally in the sensorimotor areas (precentral and postcentral gyri) with a peak electrical field strength of 0.268 V/m. As intended, the electrical fields were in phase between both hemispheres and confirmed the focal distribution over central areas (Fig. 3).

## Discussion

The goal of the present study was to determine whether tACS applied at fixed frequencies of 10 Hz and 20 Hz over sensorimotor areas can induce oscillatory aftereffects in the stimulated areas. To this end, tACS was administered bilaterally with high current densities. Compared to sham stimulation, active tACS did not modulate alpha or beta oscillations. Bayesian analyses further support the absence of effects on alpha and beta power post-tACS. Given the magnitude of the Bayes factor, it appears reasonable to assume that the present results not only represent ‘absence of evidence’, but also ‘evidence of absence’ of aftereffects.

A simple explanation for the present results is that tACS does not induce robust, reliable aftereffects in oscillatory power. This is supported by studies suggesting that tACS applied at common stimulation intensities does not induce enough intracranial current to entrain neuronal populations^56–58^. Furthermore, it has been suggested that stimulation of peripheral nerves in the skin, rather than direct neuronal stimulation, explains neuronal entrainment^59^ (see Kasten et al.^60^ for an opposite view). Even if enough current does reach cortical neurons, the absence of significant aftereffects could be explained by the high levels of inter-individual variability associated with transcranial electrical stimulation (tES^61–65^). Kasten and collaborators^60^ recently reported that three individual factors accounted for up to 87% of the variance in the strength of tACS induced aftereffects: strength of the intracranial electric field, location of the electric field with respect to the stimulation target, and match between stimulation and peak endogenous frequencies. These data suggest that individual factors such as brain anatomy have a considerable impact on the size of tACS-induced neuronal modulation^60^ and may thus significantly influence group-level effects. Individual data in the present study clearly show that significant inter-individual variability occurred in the response to both 10 Hz and 20 Hz tACS.

An alternative explanation for the present negative findings is that specific methodological factors prevented optimal stimulation effects. For example, the stimulation frequency was not matched to individual endogenous peak frequencies. For stimulation of occipital cortex, most studies have used individual alpha frequency (IAF), which resulted in predominantly significant aftereffects^24,26–28,66^ whereas fixed intensity stimulation has been less successful^29,32^. In motor cortex, fixed intensity stimulation has provided inconsistent results with some studies reporting aftereffects^47,49^ while others have not^46,48^. It is therefore possible that if tACS had been administered at individual alpha and beta frequencies in the present study, aftereffects would have been observed. However, it should be noted that using IAF does not necessarily lead to a perfect match between stimulation frequency and peak oscillatory activity. Indeed, Stecher et al.^26^ reported an average mismatch between the IAF stimulation frequency and post-stimulation alpha peak of 0.8 Hz that reached 2.5 Hz in some participants. Vossen et al.^24^ also reported that stimulation frequency and IAF can show discrepancies in the order of -1.5 Hz to 3.0 Hz. Interestingly, it was found that the strength of aftereffects is negatively correlated with the match between stimulation frequency and IAF, suggesting that a closer match between endogenous alpha oscillation peak frequency and stimulation frequency leads to weaker aftereffects^24^. More recently, Kasten and collaborators^60^ provided contradictory evidence, showing that the mismatch between IAF and stimulation frequency is a contributing factor to tACS aftereffects (less mismatch leads to greater power increases). Taken together, the available data do not provide a definitive answer as to whether stimulating at individual peak frequencies increases stimulation effects. Studies directly comparing the efficacy of fixed- and individual-intensity protocols are needed to determine whether tACS is more effective when the stimulation frequency is matched with baseline peak frequencies.

Another factor that has been shown to modulate the effects of tES is electrode size and placement, as well as stimulation intensity. Most previous studies assessing the effects of alpha or beta tACS on cortical oscillations have used at least one large stimulation electrode (35 cm^2^) with relatively low stimulation intensity (≈ 1 mA)^12,24,25,27,30,46–48^. For both EEG^49,51^ and TMS^11,51^ measures of tACS aftereffects, however, higher stimulation intensities (and current densities) have been associated with stronger effects. In the present study, tACS was applied at an intensity of 1 mA but with much smaller electrodes than what is usually used. Thus, despite using a current density that was significantly higher than what was used in most previous studies, aftereffects were not found following stimulation. One possible explanation for this is that the effect of tES intensity is non-linear. For example, increasing tDCS stimulation duration from 13 to 26 minutes^67^ or current intensity from 1 to 2 mA^68^ has been shown to reverse the direction of aftereffects. More recently, De Koninck and collaborators^69^ reported that 1 mA IAF tACS induced stronger aftereffects in alpha power than stimulation at 4–6 mA. Thus, it is possible that increasing current density had the paradoxical effect of reducing the ability of tACS to produce aftereffects in sensorimotor cortex. It should be noted, however, that similar off-line protocols to the one used in the present study (bilateral, 1 mA, 3.14 cm^2^ electrode size) have been shown to modulate brain oscillations. For example, Berger and collaborators^34^ reported that 20 min of bilateral alpha-tACS (P3-P4) increased alpha oscillations while Hsu et al.^53^ reported that bilateral theta-tACS (F3-F4) increased beta oscillations. Nevertheless, FEM data show that most of the current in the present study was distributed over the sensorimotor areas. Furthermore, despite higher current densities being delivered owing to smaller electrode surface, electrical field strengths were within the range of previously reported models, albeit near the upper limit^30,44,70^. This suggests that the stimulation protocol used in the present study was efficient in targeting sensorimotor areas with relatively high current strength.

Another feature of tES protocols that may have an effect on physiological response is the state of the brain at the time of stimulation. It has been repeatedly shown that stimulation protocols are state-dependent^71^. For example, the effects of tACS on alpha oscillations have been shown to disappear when participants have their eyes closed during the experiment, which could be explained by increased endogenous alpha activity^25^. Here again, there is variability in the studies that have assessed the aftereffects of alpha and beta tACS with regards to state-dependency. In some studies, participants were required to perform a task that was relevant to the experimental question during stimulation^29,31–34^. In other studies, a simple task was performed throughout the entire experiment (before, during, and after stimulation) to maintain vigilance levels^12,24–26,28,30^. In the present study, participants were asked to watch a documentary film during the entire experiment, as previously described^66^. It is possible that passive film viewing was not engaging enough for participants, which led to fatigue and increases in alpha power. Alpha power at baseline, however, was not correlated with the strength of the aftereffects in the alpha frequency, suggesting that pre-stimulation alpha levels were not related to the efficacy of tACS in inducing aftereffects.

In conclusion, the present data do not support the idea that administration of higher current density by using smaller electrodes leads to robust aftereffects in oscillatory activity following tACS. The important interindividual variability and selection of specific stimulation parameters may account for the negative findings. As a result, individually tailored stimulation protocols^60^ may increase the reliability and efficacy of tACS protocols and help determine what specific factors contribute to overall response.

## Methods

### Participants

Thirty healthy right-handed (Edinburgh Handedness Inventory: 90.28 ± 10.67) volunteers (20 females, 18–37 years, mean age = 23.50 ± 3.86) were recruited via public ads to take part in the experiment. Exclusion criteria were history of neurological or psychiatric disorders, history of head injury resulting in loss of consciousness, cardiac pacemaker, presence of intracranial metal implant, tinnitus, history of seizures, history of fainting, substance abuse, and other contraindications to TMS and tACS^72^. This study conformed to the standards set by the *Declaration of Helsinki* and all the procedures were approved by the *Comité d’éthique de la recherche en arts et sciences* (CÉRAS) of the *Université de Montréal*. Written informed consent was obtained from all participants.

### Procedure

**Figure 4:**
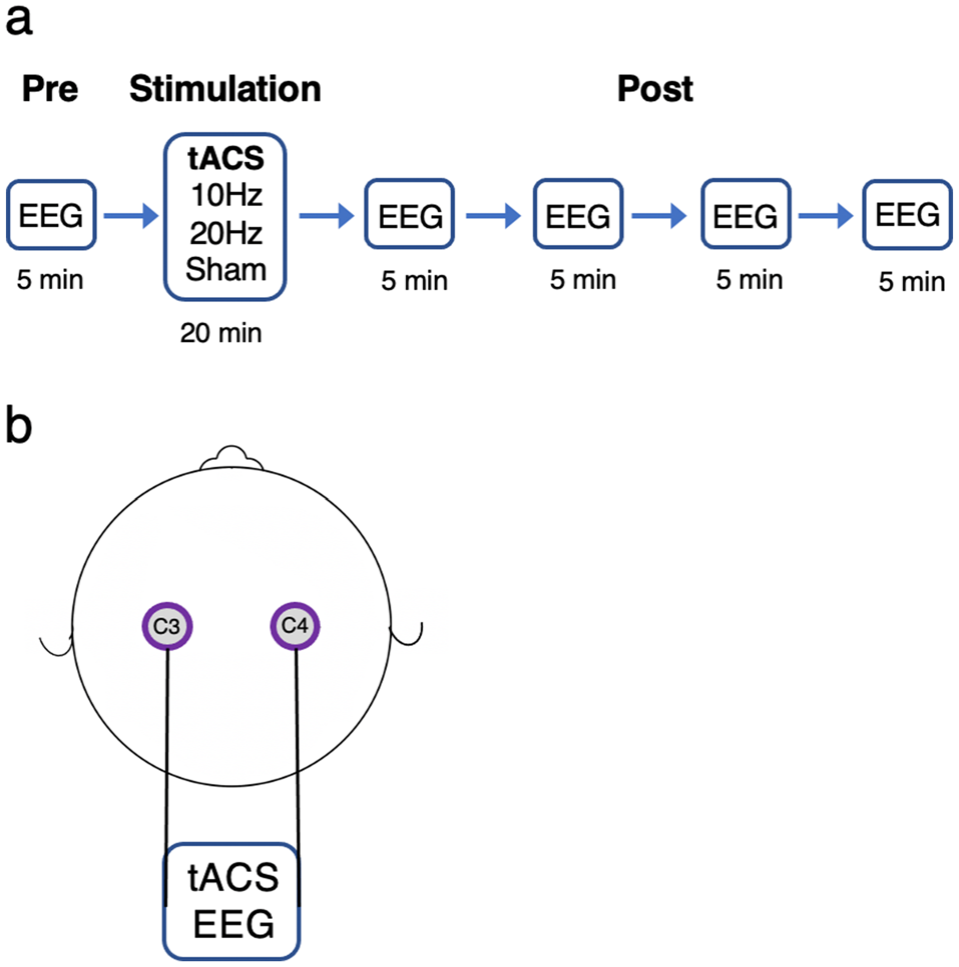
Experimental design. (**a**) Five minutes of resting-state EEG were acquired before tACS. Immediately following the end of stimulation, resting-state EEG was recorded for an additional 20 min (**b**) Stimulating and recording electrodes were positioned over C3 and C4.

In a fully within-subject, counterbalanced and double-blind design, participants took part in three experimental sessions, each separated by at least 72 h: (1) 10 Hz tACS bilateral stimulation; (2) 20 Hz tACS bilateral stimulation; (3) sham stimulation. During experimental sessions, participants were conformably seated in a chair located in an electrically shielded cabin. Electrodes were mounted on a Starstim (Neuroelectrics, Barcelona, Spain) headcap placed on the head of the participant. Once the headcap was in place, electrode gel (SignaGel, Parker Laboratories, Fairfield, USA) was applied on the contact surface between the electrode and the scalp to decrease impedance and improve conductivity. Each session lasted approximately 45 min and consisted of three blocks: (1) Baseline EEG recording for 5 min; (2) 10 Hz tACS, 20 Hz tACS or sham stimulation for 20 min; (3) EEG recording for 20 min (Fig. 4a). During the experimental sessions, participants watched an episode of the British Broadcasting Corporation’s “Planet Earth” on a computer screen located 60 cm from the participant. This was used to maintain attention without producing overt emotional response.

### tACS and EEG recordings

tACS was delivered for 20 min with a Starstim 8 tCS-EEG system (Neuroelectrics, Barcelona, Spain) at an intensity of 1 mA peak-to-peak. Hybrid tCS and EEG Pistim Ag–AgCl pellet electrodes with a 12 mm diameter and 3.14 cm^2^ circular contact area were used for stimulation with a current density of 0.32 mA/cm^2^. Stimulation was administered with two electrodes positioned on C3 and C4 (Fig. 4b). Stimulation intensity was progressively ramped up for the first 30 s of stimulation and ramped down for the last 30 s of stimulation. For sham stimulation, the current was turned off after the ramp up period for the remainder of the stimulation session. Electrode impedance was verified before each session and stimulation started only when it was below 10kΩ. The system was set up to stop stimulation if impedance exceeded 15 kΩ, which did not occur in the present experiment.

EEG was recorded using the same system and electrodes at a sampling rate of 500 Hz and analyzed offline. Signal was obtained from eight electrodes (F3, F4, Fz, C3, C4, Cz, P3, P4) mounted on a neoprene headcap in accordance with the international 10–20 EEG system. Online electrical reference earclips consisted of two opposed Ag/AgCl pellets of 8 mm diameter on the right ear.

### EEG data analysis

The offline analysis of the EEG data was performed using BrainVision Analyzer 2.2 (Brain Products GmbH, Gilching, Germany). Continuous raw data were low-pass filtered at 50 Hz (fourth-order Butterworth, zero-phase shift) and high-pass filtered at 0.5 Hz. A 60 Hz notch filter was applied to attenuate electrical interference. Data were re-referenced to electrode Fz and downsampled at 256 Hz. A 60 Hz notch filter was applied to attenuate electrical interference. EEG segments were separated into five-minute periods. The first segment corresponded to baseline (T0; pre-stimulation), and the four others to post-stimulation recordings (T1; 0–5 min, T2; 5–10 min, T3; 10–15 min, T4; 15–20 min).

All segments were then split into 1 s epochs, and segments contaminated by eye blinks or muscle movements were excluded using a semiautomatic artifact detection algorithm (min–max 100 µV criterion). A minimum number of 100 EEG clean segments per time interval for each stimulation condition was set as a criterion for a participant to be included in the analysis. No participant was excluded on this basis, with most participants having more than 250 clean segments in each time interval. For power analysis, Fast Fourier Transforms (FFT) were computed on individual epochs with 1 Hz frequency resolution using a Hanning window function (10%). Epochs were averaged for each time interval and condition and the mean power activity (µV^2^) was extracted for alpha (*α*) and beta (*β*) frequencies, corresponding to the average power between 8–12 Hz, and 13–30 Hz, respectively. Power data were log-transformed using a natural logarithm to meet the normality assumption required for analysis of variance since raw data and their residuals for the ANOVA model were not normally distributed. To avoid negative values, a value of 1 was added to all raw data before being log-transformed.

### Electrical field simulation

The SimNIBS 2 software pipeline was used for electrical field simulation^73^, based on a finite-element method (FEM) that allows for precise calculations of electric fields in complex geometrical shapes such as a human head. tACS electrical distribution was simulated under a quasi-static regime assumption^74,75^ since at relatively low frequencies of tDCS (< 1 kHz, in the present study 10 and 20 Hz) the electrical fields can be separated into spatial and temporal components. In the case of a sinusoidal current with a specific frequency and amplitude, the electric field will vary in time and with the same frequency and phase of the input current^74,75^. As a result, the electrical field obtained at peak currents can be simulated, with the temporal variations of the current scaling the field with no changes of its distribution in the brain.

The spatial component was calculated by solving Laplace's equation for the electrostatic potential φ

$$ \nabla \cdot (\upsigma \nabla {\varphi }) \, = \, 0 $$

using Dirichlet boundary conditions at the electrodes (σ representing the ohmic conductivity, and ∇ the divergence and gradient differential vector operators, respectively). The FEM solver^76^ used the Galerkin method based on tetrahedral first order elements, and the residuals for the conjugate gradient solver were required to be < 10^−9^.

The electric field vector (E) was then determined by the numerical differentiation of φ.

$$ {\text{E}} = - \nabla {\varphi } $$

The current density J was determined via Ohm's law (J = delta*E). The electrostatic potential and the field values were scaled such that a current unit (i.e., 1) was passing through the electrodes.

### Statistical analysis

Repeated measures analysis of variance (rmANOVA), with factors Condition (10 Hz, 20 Hz, sham) and Time (T0, T1, T2, T3, T4) was used separately to test for changes in *α* and *β* EEG power. This analysis was conducted for the two electrodes of interest (C3 and C4) at each of the two frequency bands. In the case of significant interaction effects, *post-hoc* analyses were performed with Bonferroni corrections to control for multiple comparisons, and non-sphericity was adjusted using Greenhouse-Weisser correction when required. To determine whether baseline alpha or beta activity is associated with the effects of tACS, Pearson’s correlation coefficients were computed with the log-transformed baseline power values and T1/T0, T2/T0, T3/T0 and T4/T0 power ratios for electrodes C3 and C4. No participant data were removed from analysis.

Additional Bayesian statistical analyses were tested with the JASP package (version 0.14.1^77^) to quantify the plausibility of alternative *H*_1_ versus the null *H*_0_ hypotheses. Bayesian repeated measures ANOVAs were conducted using JASP default priors, and effects are reported as the Bayes factor for the inclusion (BF_incl_) of a particular effect, calculated as the ratio between the likelihood of the data given the model compared to the model without that specific effect (see Keysers et al.^78^ for detailed description of BF_incl_).

## Supplementary Information

Supplementary Tables.

## Data Availability

The datasets generated during and/or analyzed during the current study are available from the corresponding author on request.
