# Supplementary material for: No aftereffects of high current density 10 Hz and 20 Hz tACS on sensorimotor alpha and beta oscillations
Source: Sci Rep. 2021 Nov 1;11:21416. doi: 10.1038/s41598-021-00850-1 (PMC8560917; doi:10.1038/s41598-021-00850-1)
Supplement: Supplementary file 1 — Supplementary Tables. [file 41598_2021_850_MOESM1_ESM.docx]

**No aftereffects of high current density 10Hz and 20Hz tACS on sensorimotor alpha and beta oscillations**

Louis-Philippe Lafleur ^1^, Audrey Murray ^2,3,4^, Manon Desforges ^5,6^, Kevin Pacheco-Sara^7^, Felipe Fregni ^7^, Sara Tremblay ^5,6^, Dave Saint-Amour ^2,3^, Jean-François Lepage ^8^, Hugo Théoret ^1,3*^

^1^ Département de psychologie, Université de Montréal, Montréal, Canada

^2^ Département de psychologie, Université du Québec à Montréal, Montréal, Canada

^3^ Centre de recherche du Centre Hospitalier Universitaire Sainte-Justine, Montréal, Canada

^4^ Centre Intégré Universitaire de Santé et de Services Sociaux du Nord-de-l’île-de-Montréal, Montréal, Canada

^5^ Département de psychoéducation et de psychologie, Université du Québec en Outaouais, Gatineau, Canada

^6^ University of Ottawa Institute of Mental Health Research at The Royal, Ottawa, Canada

^7^ Neuromodulation Center and Center for Clinical Research Learning, Spaulding Rehabilitation Hospital and Massachusetts General Hospital, Harvard Medical School, Boston, Massachusetts, USA

^8^ Département de Pédiatrie, Faculté de Médecine et des Sciences de la Santé de l’Université de Sherbrooke, Centre de recherche du CHU Sherbrooke, Sherbrooke, Canada

**Running head:** Aftereffects of tACS over sensorimotor areas

***Corresponding author:**

Hugo Théoret. PhD

Département de Psychologie

Université de Montréal

CP 6128. Succ. Centre-Ville

Montréal. QC. Canada. H3C 3J7

Tel : 514-343-6362

e-mail : hugo.theoret@umontreal.ca

**Supplementary Table S1.** Model comparison table obtained from Bayesian repeated measures ANOVA for *α*-power at electrode site C3. Results were computed using JASP program.

| **Model Comparison** | | | | | | | | | | | |
| --- | --- | --- | --- | --- | --- | --- | --- | --- | --- | --- | --- |
| **Models** | | **P(M)** | | **P(M\|data)** | | **BF _M_** | | **BF _10_** | | **error %** | |
| Null model (incl. subject) |  | 0.200 |  | 0.004 |  | 0.018 |  | 1.000 |  |  |  |
| Condition |  | 0.200 |  | 0.984 |  | 246.577 |  | 219.289 |  | 5.806 |  |
| Condition + Time |  | 0.200 |  | 0.011 |  | 0.046 |  | 2.535 |  | 1.229 |  |
| Time |  | 0.200 |  | 5.330e -5 |  | 2.132e -4 |  | 0.012 |  | 0.666 |  |
| Condition + Time + Condition  ✻  Time |  | 0.200 |  | 4.481e -5 |  | 1.793e -4 |  | 0.010 |  | 1.814 |  |
|  | | | | | | | | | | | |
|  | | | | | | | | | | | |

**Supplementary Table S2.** Effects obtained from Bayesian repeated measures ANOVA for *α*-power at electrode site C3. Relative strong evidence (BF_incl_) was found in favor of an effect of *Condition* and relative moderate evidence against a main effect of *Time*. There was relative strong evidence for the null hypothesis (*H*_0_) over the alternative (*H*_1_) for *Condition X Time* interaction.

| **Analysis of Effects** | | | | | | | | | | | |
| --- | --- | --- | --- | --- | --- | --- | --- | --- | --- | --- | --- |
| **Effects** | | **P(incl)** | | **P(excl)** | | **P(incl\|data)** | | **P(excl\|data)** | | **BF _incl_** | |
| Condition |  | 0.400 |  | 0.400 |  | 0.995 |  | 0.005 |  | 219.220 |  |
| Time |  | 0.400 |  | 0.400 |  | 0.011 |  | 0.989 |  | 0.012 |  |
| Condition  ✻  Time |  | 0.200 |  | 0.200 |  | 4.481e -5 |  | 0.011 |  | 0.004 |  |
|  | | | | | | | | | | | |
|  | | | | | | | | | | | |

**Supplementary Table S3.** Model comparison table obtained from Bayesian repeated measures ANOVA for *α*-power at electrode site C4. Results were computed using JASP program.

| **Model Comparison** | | | | | | | | | | | |
| --- | --- | --- | --- | --- | --- | --- | --- | --- | --- | --- | --- |
| **Models** | | **P(M)** | | **P(M\|data)** | | **BF _M_** | | **BF _10_** | | **error %** | |
| Null model (incl. subject) |  | 0.200 |  | 0.818 |  | 17.958 |  | 1.000 |  |  |  |
| Condition |  | 0.200 |  | 0.172 |  | 0.834 |  | 0.211 |  | 0.842 |  |
| Time |  | 0.200 |  | 0.008 |  | 0.032 |  | 0.010 |  | 1.059 |  |
| Condition + Time |  | 0.200 |  | 0.002 |  | 0.007 |  | 0.002 |  | 1.621 |  |
| Condition + Time + Condition  ✻  Time |  | 0.200 |  | 1.006e -5 |  | 4.022e -5 |  | 1.230e -5 |  | 1.120 |  |
|  | | | | | | | | | | | |

**Supplementary Table S4.** Effects obtained from Bayesian repeated measures ANOVA for *α*-power at electrode site C4. Relative moderate evidence (BF_incl_) was found against a main effect of *Condition* and *Time*. There was relative strong evidence for the null hypothesis (*H*_0_) over the alternative (*H*_1_) for *Condition X Time* interaction.

| **Analysis of Effects** | | | | | | | | | | | |
| --- | --- | --- | --- | --- | --- | --- | --- | --- | --- | --- | --- |
| **Effects** | | **P(incl)** | | **P(excl)** | | **P(incl\|data)** | | **P(excl\|data)** | | **BF _incl_** | |
| Condition |  | 0.400 |  | 0.400 |  | 0.174 |  | 0.826 |  | 0.211 |  |
| Time |  | 0.400 |  | 0.400 |  | 0.010 |  | 0.990 |  | 0.010 |  |
| Condition  ✻  Time |  | 0.200 |  | 0.200 |  | 1.006e -5 |  | 0.002 |  | 0.006 |  |
|  | | | | | | | | | | | |

**Supplementary Table S5.** Model comparison table obtained from Bayesian repeated measures ANOVA for *β*-power at electrode site C3. Results were computed using JASP program.

| **Model Comparison** | | | | | | | | | | | |
| --- | --- | --- | --- | --- | --- | --- | --- | --- | --- | --- | --- |
| **Models** | | **P(M)** | | **P(M\|data)** | | **BF _M_** | | **BF _10_** | | **error %** | |
| Null model (incl. subject) |  | 0.200 |  | 5.106e -4 |  | 0.002 |  | 1.000 |  |  |  |
| Condition |  | 0.200 |  | 0.995 |  | 750.316 |  | 1947.961 |  | 2.003 |  |
| Condition + Time |  | 0.200 |  | 0.005 |  | 0.019 |  | 9.351 |  | 0.986 |  |
| Condition + Time + Condition  ✻  Time |  | 0.200 |  | 1.492e -5 |  | 5.966e -5 |  | 0.029 |  | 1.248 |  |
| Time |  | 0.200 |  | 2.460e -6 |  | 9.842e -6 |  | 0.005 |  | 0.502 |  |
|  | | | | | | | | | | | |

**Supplementary Table S6.** Effects obtained from Bayesian repeated measures ANOVA for *β*-power at electrode site C3. Relative strong evidence (BF_incl_) was found in favor of a main effect of *Condition*. There were relative strong evidences supporting null hypothesis (*H*_0_) compared to *H*_1_ for the main effect of *Time*, and for the *Condition X Time* interaction.

| **Analysis of Effects** | | | | | | | | | | | |
| --- | --- | --- | --- | --- | --- | --- | --- | --- | --- | --- | --- |
| **Effects** | | **P(incl)** | | **P(excl)** | | **P(incl\|data)** | | **P(excl\|data)** | | **BF _incl_** | |
| Condition |  | 0.400 |  | 0.400 |  | 0.999 |  | 5.131e -4 |  | 1947.926 |  |
| Time |  | 0.400 |  | 0.400 |  | 0.005 |  | 0.995 |  | 0.005 |  |
| Condition  ✻  Time |  | 0.200 |  | 0.200 |  | 1.492e -5 |  | 0.005 |  | 0.003 |  |
|  | | | | | | | | | | | |

**Supplementary Table S7.** Model comparison table obtained from Bayesian repeated measures ANOVA for *β*-power at electrode site C4. Results were computed using JASP program.

| **Model Comparison** | | | | | | | | | | | |
| --- | --- | --- | --- | --- | --- | --- | --- | --- | --- | --- | --- |
| **Models** | | **P(M)** | | **P(M\|data)** | | **BF _M_** | | **BF _10_** | | **error %** | |
| Null model (incl. subject) |  | 0.200 |  | 0.071 |  | 0.305 |  | 1.000 |  |  |  |
| Condition |  | 0.200 |  | 0.925 |  | 49.203 |  | 13.070 |  | 0.617 |  |
| Condition + Time |  | 0.200 |  | 0.004 |  | 0.016 |  | 0.058 |  | 1.042 |  |
| Time |  | 0.200 |  | 3.132e -4 |  | 0.001 |  | 0.004 |  | 0.658 |  |
| Condition + Time + Condition  ✻  Time |  | 0.200 |  | 1.499e -5 |  | 5.995e -5 |  | 2.118e -4 |  | 1.546 |  |
|  | | | | | | | | | | | |

**Supplementary Table S8.** Effects obtained from Bayesian repeated measures ANOVA for *β*-power at electrode site C4. Relative strong evidences (BF_incl_) were found in favor of a main effect of *Condition,* and against a main effect of *Time*. There was relative strong evidence supporting null hypothesis (*H*_0_) over *H*_1_ for *Condition X Time* interaction.

| **Analysis of Effects** | | | | | | | | | | | |
| --- | --- | --- | --- | --- | --- | --- | --- | --- | --- | --- | --- |
| **Effects** | | **P(incl)** | | **P(excl)** | | **P(incl\|data)** | | **P(excl\|data)** | | **BF _incl_** | |
| Condition |  | 0.400 |  | 0.400 |  | 0.929 |  | 0.071 |  | 13.070 |  |
| Time |  | 0.400 |  | 0.400 |  | 0.004 |  | 0.996 |  | 0.004 |  |
| Condition  ✻  Time |  | 0.200 |  | 0.200 |  | 1.499e -5 |  | 0.004 |  | 0.004 |  |
|  | | | | | | | | | | | |

**Supplementary Table S9.** Summary table of the relative evidence in favor of both alternative *H*_1_ or null *H*_0_ hypotheses using the qualitative description of the JASP guidelines classification.

|  | ***α*-power** | | ***β*-power** | |
| --- | --- | --- | --- | --- |
| **Electrode** | **C3** | **C4** | **C3** | **C4** |
| Condition | strong *H*_1_ | moderate *H*_0_ | strong *H*_1_ | strong *H*_1_ |
| Time | moderate *H*_0_ | moderate *H*_0_ | strong *H*_0_ | strong *H*_0_ |
| Condition Time | strong *H*_0_ | strong *H*_0_ | strong *H*_0_ | strong *H*_0_ |
|  | | | | |
|  | | | | |
